# Electrochemical Oxidation of Monosaccharides at Nanoporous Gold with Controlled Atomic Surface Orientation and Non-Enzymatic Galactose Sensing

**DOI:** 10.3390/s20195632

**Published:** 2020-10-01

**Authors:** Yasuhiro Mie, Shizuka Katagai, Masiki Ikegami

**Affiliations:** Bioproduction Research Institute, National Institute of Advanced Industrial Science and Technology (AIST), Sapporo 062-8517, Japan; s.katagai@aist.go.jp (S.K.); m.ikegami@aist.go.jp (M.I.)

**Keywords:** nanoporous gold, crystallographic orientation, monosaccharide oxidation, galactose sensor

## Abstract

Non-enzymatic saccharide sensors are of great interest in diagnostics, but their non-selectivity limits their practical diagnostic abilities. In this study, we investigated the electrochemical oxidation of monosaccharides at nanoporous gold (NPG) catalysts with different contributions of surface crystallographic orientations. Fructose elicited no clear electrochemical response, but glucose, galactose, and mannose produced clear oxidative current. The onset potentials for oxidation of these saccharides depended on the surface atomic structure of the NPG. The oxidation potential was approximately 100 mV less positive at the Au(100)-enhanced NPG than at the Au(111)-enhanced NPG. Furthermore, the voltammetric responses significantly differed among the saccharides. Galactose was oxidized at less positive potential and exhibited a higher current response than the other saccharides. This tendency was enhanced in the presence of chloride ions. These features enabled the selective and sensitive detection of galactose at an NPG electrode without enzymes under physiological conditions. A linear range of 10 μM to 1.8 mM was obtained in the calibration plot, which was comparable to those in previously reported enzymatic galactose sensors. Thus, we demonstrated that controlling the crystallographic orientation on the nanostructured electrode surface is useful in developing electrochemical sensors.

## 1. Introduction

Naturally occurring sugars such as glucose and galactose are involved in many biological processes. The accurate detection of these monosaccharides is critically important in clinical chemistry, food science, human nutrition, and fermentation industries [1,2]. Diabetes mellitus is a chronic and serious disease caused by disorders in carbohydrate metabolism, and characterized by abnormally high blood glucose levels. Determining glucose level is important for the diagnosis and treatment of diabetes. Elevated galactose levels are also symptomatic of certain diseases such as galactosemia, galactosuria, and other metabolic disorders [3,4]. Galactosemia is a genetically inherited metabolic disorder caused by the absence of enzyme involved in galactose metabolism. This disease is normally treated by restricting dietary galactose. As dairy products are the most common food sources of galactose, they should be avoided by patients with galactosemia [5]. In normal adults, the galactose concentration should be less than 0.28 mM. For neonates less than 5 days old, if the galactose concentration in the blood exceeds 1.11 mM, galactosemia can be fatal [6]. Thus, determination of galactose is also significant in diagnostics.

Levels of saccharide (especially glucose) can now be measured by simple and rapid methods on various sensing platforms. The electrochemical method is well-suited to this purpose [7,8,9,10]. Enzymes are frequently used as sensing agents owing to their excellent selectivity, and their activity, selectivity, and stability/durability have been improved by enzyme engineering [11,12,13]. On the other hand, non-enzymatic electrochemical sensing for glucose has been reported, where glucose is directly oxidized at the electrode surface [14,15,16,17,18], although that for galactose is very limited. This strategy resolves the stability issues of enzymes stemming from their intrinsic properties. Electrodes composed of nanomaterials have been selected for non-enzymatic glucose oxidation because the nanostructures increase the number of lowly coordinated surface atoms, and hence their electrocatalytic activity [19,20,21,22,23]. There has been increasing interest in using nanoporous gold (NPG) in electrocatalysis as an electrode for applications such as sensors and reactors owing to its high conductivity, chemical inertness, physical stability, and large surface area [24]. Glucose oxidation studies using NPG electrodes have also been reported for its sensing applications [20,21]. Improvements in various electrocatalytic reactions including glucose oxidation have been revealed using NPG [20,21,24,25,26], indicating its efficacy and potency. However, selectivity in sensing application is generally limited. On the other hand, the atomic surface structure (i.e., the surface crystallographic orientation) is another important determinant of the catalytic activity of an electrode, and the suitable orientation differs depending on the target substances [27,28,29]. However, the effect of the surface orientation in the NPG structure on electrochemical saccharide oxidation has not been discussed. Fairly recently, we reported a facile method able to control the composition of the surface crystallographic orientations of NPG catalysts [30].

**Figure 1:**
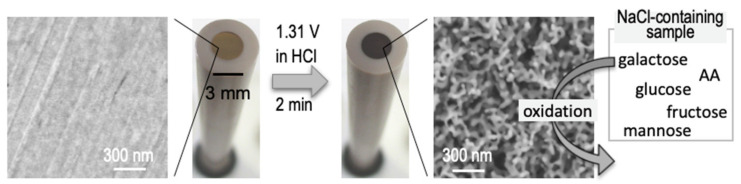
Schematic representation of NPG (right) fabrication from a commercially available gold electrode (left) for galactose sensing in this study. AA stands for ascorbic acid.

In the present study, we examined the electrochemical oxidation of monosaccharides at two kinds of NPG with different compositions of surface crystallographic orientations, and found that their oxidation behaviors depended on the surface atomic structure. Furthermore, the electrochemical responses significantly differed among the saccharides. Based on these characteristics, we developed a selective and non-enzymatic galactose sensing platform (Figure 1).

## 2. Materials and Methods

### 2.1. Materials and Chemicals

A gold disk, Pt wire, and Ag|AgCl|saturated KCl electrodes were purchased from Bioanalytical Systems Inc. (West Lafayette, IN, USA). D-glucose, D-galactose, D-mannose, D-fructose, ascorbic acid, and dialysis membrane (size 36) were obtained from FUJIFILM Wako Pure Chemical Co. (Osaka, Japan). Human serum was purchased from Sigma-Aldrich (St. Louis, MO, USA). All reagents were of the best available grade and were used as received with no further purification. All solutions were prepared with purified water (Millipore, 18 MΩ cm^−1^).

### 2.2. Preparation of NPG with Different Facet Contributions

NPG was prepared by simply applying an anodization potential as previously reported [30]. A gold disk electrode (ø = 3 mm) was polished with 1.0 and 0.125 μm of diamond slurries followed by a 0.05 μm of alumina slurry until a clear mirrorlike surface was obtained. The polished electrode was electrochemically cleaned in 0.5 M H_2_SO_4_ solution at potentials between −0.2 to 1.5 V (vs. Ag|AgCl). The cleaned electrode was placed in a 35 or 500 mM HCl solution, then anodized under an appropriate potential to fabricate a nanoporous structure. Before anodization, the passivation potential of the electrode was evaluated by linear sweep voltammetry. The gold surface had been passivated by the formation of an oxide layer, as indicated by a sudden current drop [30,31,32]. Referring to the literature, the anodization potential for NPG construction was determined as 10–40 mV more negative than the passivation potential [30,31,32]. The morphology of the NPG surface was characterized by scanning electron microscopy (SEM) (S-4300 FE-SEM, Hitachi Ltd., Tokyo, Japan). The electrochemical (actual) surface area was evaluated from the reductive charge of gold oxides in cyclic voltammograms obtained in 0.5 M H_2_SO_4_ [33]. The roughness factor (*R*_f_) was calculated by dividing the electrochemical surface area by the geometrical area of the gold disk electrode. The *R*_f_ values of NPG electrodes were arranged to be around 6 by controlling the anodization time.

### 2.3. Electrochemical Measurements

All electrochemical experiments were carried out using an ALS model 660D electrochemical analyzer (CH Instruments Inc., Austin, TX, USA) with a standard three-electrode configuration. The working, counter, and reference electrodes in this configuration were NPG, Pt wire, and Ag|AgCl|saturated KCl, respectively. All potentials were represented versus Ag|AgCl|saturated KCl. All saccharide measurements were conducted in a 0.1 M phosphate buffer solution (pH 7.5) in the presence or absence of 0.1 M NaCl at 25 °C. The cyclic voltammetric responses of the monosaccharides were obtained at monosaccharide concentrations between 1 and 10 mM. The solution was bubbled with Ar (99.999%) before each measurement. Amperometry was conducted at a certain potential while stirring the solution. The current densities of the saccharides at the NPG electrode were obtained by dividing the observed current by the electrochemical (actual) surface area (calculated as stated above). In the recovery test, the serum sample was diluted 100 times and spiked with three concentrations of galactose. The amperometric response was recorded at the NPG electrode covered with a dialysis membrane.

## 3. Results and Discussion

### 3.1. Effect of Surface Crystallographic Orientation of NPG on Glucose Electrochemistry

**Figure 2:**
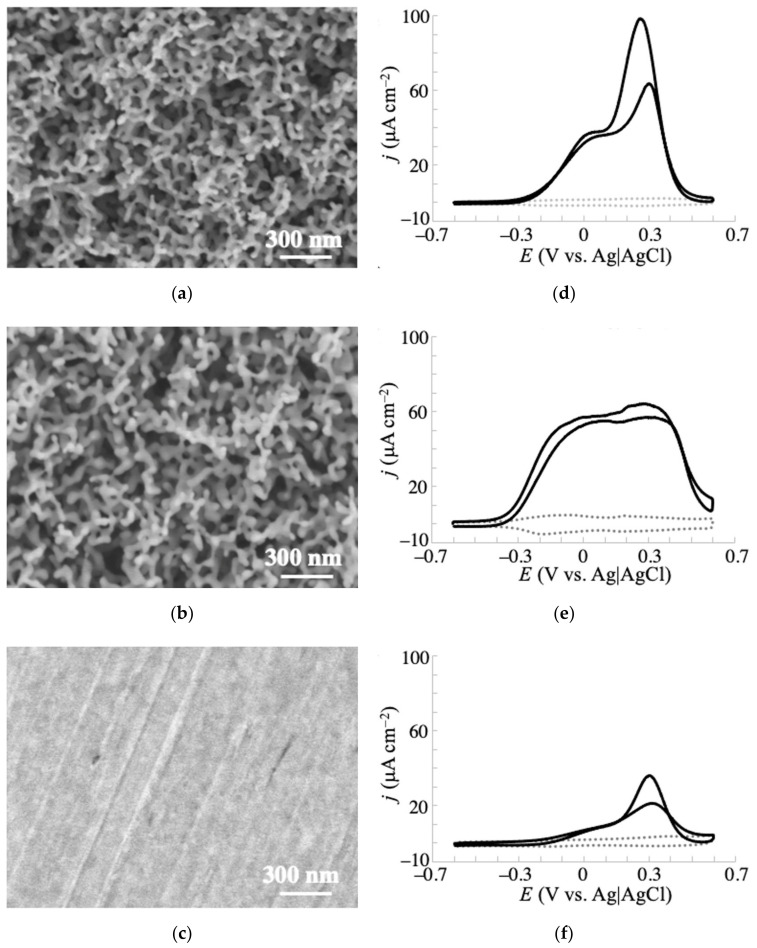
Surface morphologies of (**a**) NPG-35, (**b**) NPG-500, and (**c**) planar (non-anodized) gold electrodes observed by SEM, and (**d**–**f**) their corresponding voltammograms of 10 mM glucose in 0.1 M phosphate buffer (pH 7.5) obtained at 20 mV s^−1^. Dotted lines were the voltammograms obtained without glucose.

Previously, we reported that NPG electrodes prepared from solutions of 35 and 500 mM HCl (NPG-35 and NPG-500) exhibit different contributions of surface atomic structure and crystallographic orientation [30]. The NPG-35 and NPG-500 electrodes were enriched in the Au(111) and Au(100) orientations, respectively. The different concentrations of HCl gave different passivation potentials and hence anodization potentials (Figure S1), which led to differentiable dissolution rates of the deposited gold atoms by disproportionation of AuCl_2_^−^ in the anodization process. The formation of different contributions of the facets in NPG was suggested to result from the different reorganization time to form thermally stable Au(111) facets from the less stable facets, such as Au(100), in deposited atoms. To investigate the effects of surface crystallographic orientation on the electrochemical oxidation properties of glucose, we conducted voltammetry tests at NPG-35 and NPG-500. Figure 2a,b shows the morphologies of the NPG-35 and NPG-500 electrodes, respectively, along with the corresponding cyclic voltammograms (Figure 2d,e) of 10 mM glucose in 0.1 M phosphate buffer (pH 7.5). Figure 2c,f shows the morphology and cyclic voltammogram of the planar (non-anodized) gold electrode. The morphologies of both NPGs were composed of similarly sized ligaments (~50 nm) and nanoporous structures spread homogeneously in wide regions (Figure S2). The atomic force microscopy (AFM) image of NPG is also shown in Figure S3. Despite their similar morphologies, the two NPGs produced significantly different voltammetric responses to glucose oxidation. NPG-35 exhibited two main anodic peaks, one around 0 V and the other around 0.3 V. The shape of the voltammetric curve resembled that of the planar gold electrode, but the current density was approximately three times larger and the onset oxidation potential was approximately 50 mV less positive than those of the planar electrode. NPG-500 also yielded a multipeak voltammogram with a larger current density than planar gold. It is important to note that the onset potential of NPG-500 was less positive (by more than 100 mV) than those of NPG-35 and planar electrodes. These results indicate that the NPG structure increases electrocatalytic activity for glucose oxidation, and that the oxidation potential depends on the crystallographic orientation of the NPG surface.

Hsiao et al. studied electrochemical oxidation of glucose using single crystal gold surfaces and clarified that the crystallographic orientation significantly affects the oxidation current density and the onset potential [34]. The voltammograms of single-crystal Au(111) and Au(100) reportedly show one main peak, which is approximately 120 mV less positive for Au(100) than for Au(111). In general, an NPG structure cannot form a monolithic surface orientation because it lacks high-index facets and surface steps/kinks that maintain the curvature of its three-dimensional bicontinuous porosity [35]. NPG structures with different contributions of low-index facets have been prepared by the anodization method described above [30] and other dealloying methods [35]. Thus, the multipeaks observed in the voltammograms of glucose oxidation at NPG-35 and NPG-500 are reasonable. Furthermore, the less positive onset potential at NPG-500 with an Au(100)-enhanced surface than at NPG-35 with an Au(111)-enhanced surface is consistent with observations of single-crystal electrodes [34]. With its highly negative onset potential and high catalytic activity, NPG-500 is more promising in practical applications than NPG-35, because it should be less vulnerable to interference by easily oxidizable substances in body fluids such as blood and urine. Thus, controlling the surface atomic structure can effectively improve the catalytic properties of a nanomaterial-based electrode. The voltammetric shape of glucose catalysis at NPG-500 resembles that of a previous report, in which the NPG electrode was prepared by anodization in 1 M KCl solution [36]. This similarity suggests similar surface orientations of the NPG electrodes in [36] and the present study to some extent.

### 3.2. Electrochemistry of Monosaccharides at the NPG Electrode

**Figure 3:**
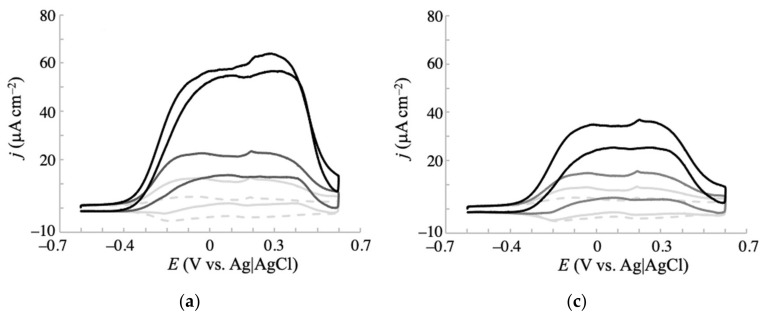

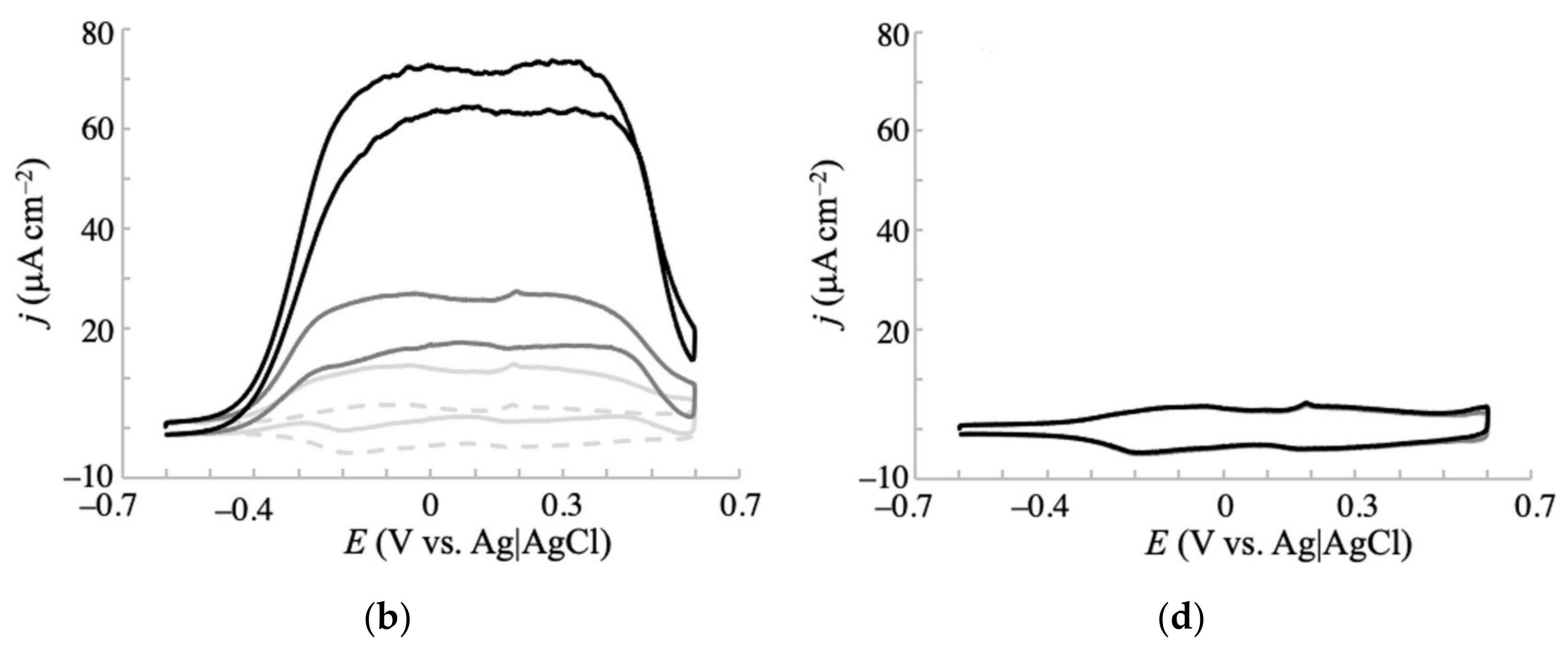
Cyclic voltammograms of (**a**) glucose, (**b**) galactose, (**c**) mannose, and (**d**) fructose in 0.1 M phosphate buffer (pH 7.5) obtained at an NPG-500 electrode with a scan rate of 20 mV s^−1^. Concentrations of each saccharide: 0 mM (dashed gray line), 1 mM (solid light gray line), 3 mM (solid gray line), and 10 mM (solid black line).

**Figure 4:**
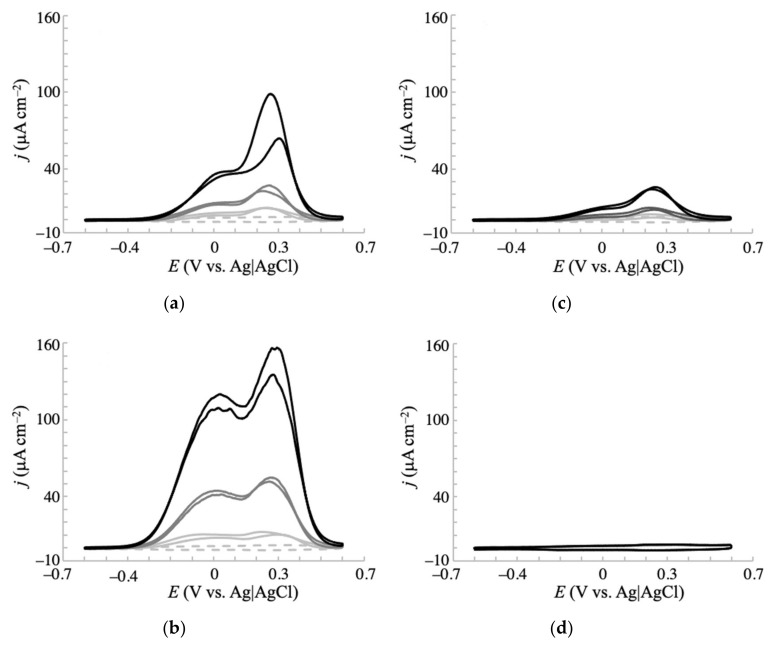
Cyclic voltammograms of (**a**) glucose, (**b**) galactose, (**c**) mannose, and (**d**) fructose in 0.1 M phosphate buffer (pH 7.5) obtained at an NPG-35 electrode with a scan rate of 20 mV s^–1^. Concentrations of each saccharide: 0 mM (dashed gray line), 1 mM (solid light gray line), 3 mM (solid gray line), and 10 mM (solid black line).

Above, we confirmed the improved electrochemical responses for glucose oxidation at the fabricated NPG electrodes. Here, we investigate the electrochemistry of other monosaccharides (galactose, mannose, and fructose) at the electrodes. Figure 3 and Figure 4 show the voltammetric responses of NPG-500 and NPG-35, respectively, to glucose and the additional monosaccharides at concentrations of 1, 3, and 10 mM in 0.1 M phosphate buffer solution (pH 7.5). In the presence of fructose (even at 10 mM), the voltammetric curves of the NPG-500 (Figure 3d) and NPG-35 (Figure 4d) electrodes were almost flat, resembling that of blank buffer solution. Such almost inactive electrochemical oxidation for fructose was previously reported for a gold single-crystal electrode [34]. Meanwhile, other saccharides showed a clear, concentration-dependent oxidation current in the voltammogram. Similar to the glucose case described above, the oxidation currents of galactose and mannose appeared at a less positive potential at NPG-500 than at NPG-35 (Figure 3b,c and Figure 4b,c), again confirming the effect of the atomic surface structures. Significantly, the oxidation current density and onset potential differed among the glucose, galactose, and mannose oxidations. For clarity, the relationship between the concentration and current density for saccharides is summarized in Figure S4. The current density induced by 10 mM saccharide at NPG-500 and −0.2 V increased in the order of galactose > glucose > mannose. The galactose and glucose exhibited 3.5 and 1.9 times larger current densities than that of mannose. The negativity of the onset potentials followed the same order as the oxidation current density. Moreover, the less inhibition by Cl^−^ at NPG-500 compared with NPG-35 was observed as described below, indicating that electrochemical oxidation at NPG-500 was maximized for galactose. Therefore, the NPG-500 electrode surface is expected to enable selective and sensitive detection of galactose.

Electrochemical oxidation of glucose has been extensively reported at various electrodes. Glucose oxidation at noble metal electrodes is suggested to occur through interactions between the hemiacetal group of glucose and the incipient hydrous oxide on the electrode surface, which mediates the reaction [1,14,34,37]. First, the hydrogen bound to the C1 carbon atom is dehydrogenated with one electron transfer. This process is the rate-determining step, as revealed by the isotope effect. Then, the resultant radical species is further oxidized to form gluconolactone. Xia et al. reported that the voltammograms of NPG electrodes have multicoupling peaks between −0.3 and 0.3 V (vs. saturated calomel electrode (SCE)) in a phosphate buffer solution, which correspond to chemisorption/desorption of OH^−^ or H_2_O to form AuOH [31]. Similar peaks were observed for the present NPG-500 as shown in Figure 2e (dotted gray line), suggesting that a similar mechanism occurs at NPG-500. NPG-35 also exhibited such peaks in the buffer; however, the ratio of those peak currents (i.e., shape of the voltammogram) was different from that of NPG-500 (dotted gray lines in Figure 2d,e). The peak current at less positive potential (around −0.2 V) was larger for NPG-500 than NPG-35. This would be a reason for the different voltammetric behaviors for the oxidation of saccharides at NPG-35 and NPG-500. The pH dependence of the galactose voltammogram also coincides with the aforementioned mechanism. With increasing pH, the oxidation current density increased (Figure S5). Such an effect was reported for electrochemical oxidation of glucose at a gold electrode [38], where it was promoted at higher pH due to more favorable chemisorption of hydroxide ions, facilitating the adsorption of glucose on the Au surface and resulting in reduced activation energy for oxidation of glucose. The aforementioned mechanism is further supported by no clear electrochemical response to the oxidation of fructose, which lacks a hydrogen atom bound to the C1 atom. According to the mechanism, the electrochemical oxidation favors the hydrogen axially bound to C1 over the equatorially bound hydrogen [1,14]. Considering the predominance of the β-anomer for galactose and glucose and the α-anomer for mannose [39], the larger electrochemical oxidation responses of glucose and galactose (that possess an axial hydrogen at the C1 atom) than of mannose (with an equatorial hydrogen atom at the same position) additionally fit the aforementioned mechanism. However, this mechanism cannot explain the more efficient electrochemical oxidation of galactose than glucose, as these molecules possess similar structures of their hemiacetal group. Judging from the different conformations of the hydrogen and hydroxy groups at the C4 atom of these monosaccharides, this hydrogen or hydroxy group might be involved in the interaction with the electrode surface. The results indicate that this interaction favors galactose. The detailed explanation of this observation is under investigation.

### 3.3. Effect of Cl^–^ Ions on the Electrochemistry of Monosaccharides at the NPG Electrode

**Figure 5:**
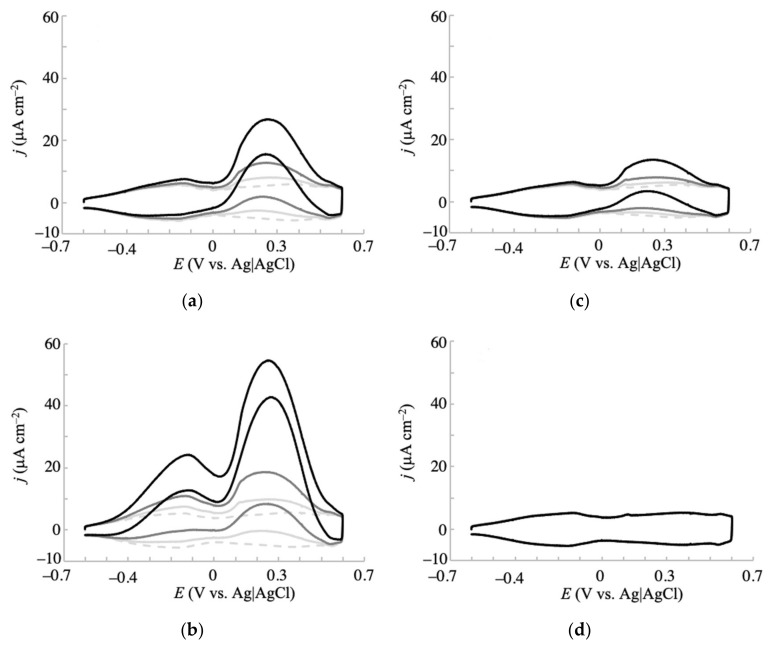
Cyclic voltammograms of (**a**) glucose, (**b**) galactose, (**c**) mannose, and (**d**) fructose in 0.1 M phosphate buffer (pH 7.5) containing 0.1 M NaCl. Voltammograms were obtained at an NPG-500 electrode with a scan rate of 20 mV s^−1^. The saccharide concentrations were varied as 0 mM (dotted gray line), 1 mM (solid light gray line), 3 mM (solid gray line), and 10 mM (solid black line).

Biological fluids are typified by large concentrations of Cl^−^ (100 mM in blood samples). As the intended application of the proposed sensors is biological sampling, suppression of the saccharide oxidation current by Cl^−^ adsorption to the gold surface is a major concern [27,36]. To investigate how Cl^−^ affects the electrochemical response to saccharide oxidation at NPG-500, the voltammetric measurements were repeated in a phosphate buffer containing 0.1 M NaCl. Figure 5 displays the obtained voltammograms of each saccharide at different concentrations (1, 3, and 10 mM). As observed in the absence of Cl^−^, fructose yielded no faradaic currents under the present conditions, reconfirming its inactiveness for electrochemical oxidation. The other saccharides delivered a lower oxidation current than in the absence of Cl^−^ (see Figure 3). This observation is consistent with previous reports on the electrochemical oxidation of glucose [27,36]. In the lower potential region (around −0.2 V), the current density of glucose and mannose decreased by more than 95% from their levels in the absence of Cl^−^. Importantly, the corresponding decrease in current density at this potential for galactose was approximately 65%, and the oxidation peak remained prominent. These results not only support the preferential interaction of the electrode surface with galactose over glucose, but also imply that the NPG-500 electrode selectively and non-enzymatically detects galactose under physiological conditions. The Cl^−^ effect on electrochemical response of galactose at NPG-35 was also conducted (Figure S6). Comparing voltammograms with and without Cl^−^ clearly showed much larger inhibition of the oxidation current density compared with that of NPG-500 electrode, indicating that NPG-500 is favorable to sensitive galactose detection under physiological conditions.

### 3.4. Non-Enzymatic Detection of Galactose With Ammperometry and Interference Effects

**Figure 6:**
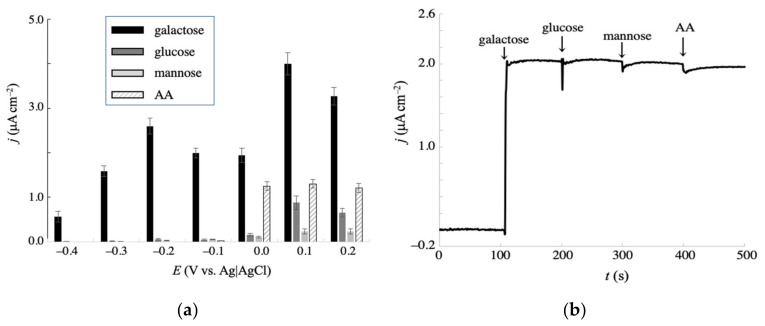
(**a**) Effect of applied potentials on the amperometric oxidative currents at NPG-500 for 2 mM galactose, glucose, mannose, and 0.2 mM AA in a 0.1 M phosphate buffer (pH 7.5) containing 0.1 M NaCl. Error bars indicate the standard deviations of triplicate independent measurements. (**b**) Representative amperogram obtained at −0.1 V under the same conditions as (**a**), with successive addition of 2 mM galactose, 2 mM glucose, 2 mM mannose, and 0.2 mM AA, indicated by the arrows at 100 s intervals.

The efficacy of NPG-500 for galactose detection was assessed in a practical amperometry study. First, we investigated the effect of applied potential on the amperometric anodic current density. Figure 6a shows the observed current densities for 2 mM glucose, galactose, mannose, and 0.2 mM ascorbic acid (AA, the major interference in blood), in the presence of 0.1 M NaCl at different potentials. Galactose detection was hardly interfered by the other saccharides and AA under applied potentials between −0.1 and −0.4 V, indicating its non-enzymatic and selective detection by NPG-500. At more positive potentials, the current densities of glucose and AA considerably increased, and obviously interfered with the galactose signals. Lower potentials reduced the interference effect, as expected. Figure 6b shows a representative amperogram obtained at −0.1 V. After galactose addition, the current increased and reached a steady state within 10 s. The stable current response after successive addition of 2 mM saccharides confirmed no significant fouling on the electrode surface. To further confirm the interference effect, amperometry with altered addition of analytes was performed. Figure S7 shows the amperogram obtained at the same conditions in Figure 6b except for the sequence of addition. While the lastly added galactose exhibited a prominent current signal, it was less from glucose, mannose, and AA. Besides, the repeated injection of galactose showed a similar current increase. These coincide with the prominent response for galactose at NPG-500 in the presence of 0.1 M NaCl, as described above.

**Figure 7:**
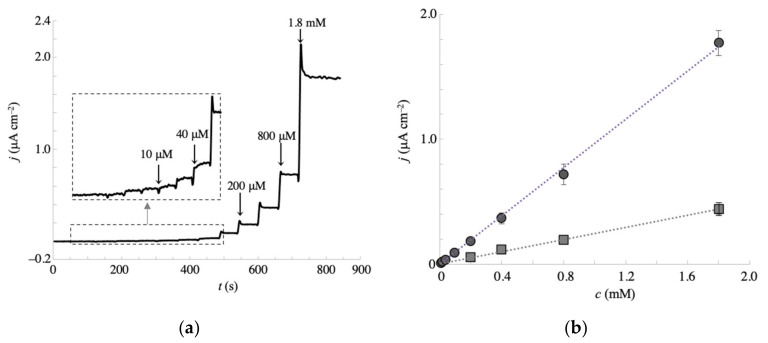
(**a**) Amperometric response of the NPG-500 electrode to successive additions of galactose in a stirred solution of 0.1 M phosphate buffer (pH 7.5) containing 0.1 M NaCl. Measurements were taken at −0.1 V. The inset is an enlargement of the response in the lower concentration region. (**b**) Calibration curve obtained from the amperograms at NPG-35 (square) and NPG-500 (circle), relating the relationship between current intensity and galactose concentration. Error bars indicate the standard deviations of triplicate independent measurements.

To prepare a calibration plot for galactose detection, we performed amperometric measurements at −0.1 V, of which potential showed relatively lower standard deviations, less interference effect, and higher current density, with successive additions of galactose at different concentrations. The amperogram and calibration curve are presented in Figure 7a,b, respectively. The calibration plot was linear from 10 μM to 1.8 mM, with a sensitivity of 1.0 μA cm^−2^ mM^−1^ and a correlation coefficient of 0.998. The limit of detection (LOD) was estimated to be 5.0 mM. To assess the analytical performance of the present sensing system, the characteristic parameters such as LOD, linear range, sensitivity, response time, working potential, and enzyme requirement were compared with earlier electrochemical galactose sensors (Table 1). Although the sensitivity is lower compared to some enzymatic sensors, the other parameters are comparable and less positive working potential enabling less interference effects was achieved. Thus, the potency of the present non-enzymatic type galactose sensor was indicated because it has an advantage of no requirements of the enzyme reagents such as galactose oxidase and dehydrogenase.

The calibration curve at the NPG-35 electrode was also plotted in Figure 7b (square symbol) to show the importance in surface orientation. The LOD and sensitivity were 100 μM and 0.2 μA cm^−2^ mM^−1^, respectively. Thus, the superiority of NPG-500 compared with NPG-35 was clearly shown under the physiological conditions, indicating the efficacy of control for the surface crystallographic orientation in NPG structure to develop efficient electrochemical sensors. To clarify the maintenance for the predominant crystallographic orientation at the NPG-500 surface, the voltammetric responses in H_2_SO_4_ solution before and after seven amperometric measurements of galactose were investigated (Figure S8A). The oxidation (oxide film formation) peak potential of the gold surface is known as an indicator for the low-index crystal planes, Au(111), Au(100), and Au(110) [49]. The voltammograms obtained showed a larger peak at 1.15 V, which corresponds to the Au(100) surface, and were almost identical before and after amperometric measurements, revealing the predominant crystallographic orientation remains during saccharide sensing. This result also suggests the repeatable use of the present NPG electrode. The reproducibility is another key index for the electrode system to serve as a promising sensor. To investigate this, the current responses of five different electrodes in amperometry to 2 mM galactose in 0.1 M phosphate buffer (pH 7.5) containing 0.1 M NaCl (triplicate measurements of each electrode) were compared (Figure S8B) and the relative standard deviation was estimated to be only 4.2%.

### 3.5. Recovery Test of Galactose in a Serum Sample

The practical applicability of the present method was assessed in recovery tests of a human serum sample spiked with standard galactose solution. The human serum was diluted by a factor of 100 and the NPG electrode was covered by a dialysis membrane to minimize fouling of its surface and/or decomposition of the spiked galactose. The serum was spiked with galactose at three concentrations (10, 100, and 1000 μM) and the amperometric measurements were conducted at the NPG-500 electrode. The obtained current was compared with that obtained in the buffered solution (calibration curve), and the recovery was determined from the current ratio. The galactose recovery was 86–101% and the relative standard deviations of the three replicated measurements were below 7.9% (Table 2). This result indicates the reliability and efficacy of the present surface-controlled NPG electrode for galactose sensing. It should be noted that when the blood sample with or without dilution (e.g., 100 times) is applied directly to the present sensor system to achieve rapid analysis without other procedures, improvement in linear range property or in inhibition of the electrode surface fouling should be required.

## 4. Conclusions

In this report, we investigated the electrochemical oxidation of monosaccharides at NPG electrodes with controlled contributions of their surface crystallographic orientations. The efficacy of the saccharide reactions was higher at the Au(100)-enhanced NPG surface than at the Au(111)-enhanced NPG surface. Interestingly, the electrochemical properties of the monosaccharides significantly differed at the NPG electrode, and the oxidative reaction was more pronounced for galactose than the other saccharides, enabling the non-enzymatic selective detection of galactose. The present results indicate that the crystallographic orientation control of nanostructured materials can achieve efficient catalysis for electrochemical reactions. Since the present method to control surface crystallographic orientation is facile, it would be useful to develop effective electrodes for sensing applications.

## Figures and Tables

**Table 1:** Comparison of characteristics for various amperometric galactose sensors.

Electrode	Enzyme	LOD (µM)	Linear Range (mM)	Sensitivity	Response Time (s)	Working Potential (V) ^a^	Ref.
1,3-DAB|Res|Pt	GalOx	50.0	0.05–6.0	-	18	+0.7	[40]
Co_3_O_4_|Graphene|GCE	GalOx	3.0	0.009–0.6	6.6 µA mM^−1^ cm^−2^	15	+0.7	[41]
Co_3_O_4_|MWCNTs|GCE	GalOx	0.9	0.009–1.0	10.4 µA mM^−1^ cm^−2^	15	+0.7	[41]
PEG|Polyanion|Pt	GalOx	-	0.0–24.0	106 nA mM^−1^ cm^−2^	<40	+0.4	[42]
PEP|Au	GalOx	25.0	2.0–16.0	1.8 µA mM^−1^	5	+0.7	[43]
Laponite clay|Pt	GalOx	1.0	0.001–1.6	85.0 mA mM^−1^ cm^−2^	5	+0.6	[44]
NADP^+^|Os|CPE	GADH	200	1.0–3.0	1.7 µA mM^−1^ cm^−2^	-	+0.15	[45]
CHIT|PGE	GalOx	50.0	0.05–25	7.0 µA mM^−1^ cm^−2^	2	+1.1	[46]
Microtubeles|ITO	GalOx	10	0.1–1.0	6.37 µA mM^−1^ cm^−2^	30–40	+0.60	[47]
CHIT|SWCNT|GCE	GalOx	25	Up to 1.0	1126 nA mM^−1^ cm^−2^	-	−0.4	[48]
NPG	none	5.0	0.01–1.8	1.0 µA mM^−1^ cm^−2^	10	−0.1	This work

^a^ V vs. Ag|AgCl. *1,3-DAB* 1,3-diaminobenzene, *Res* resorcinol, *GalOx* galactose oxidase, *MWCNTs* multiwalled carbon nanotubes, *PEG* poly(ethylene glycol), *PEP* poly(N-glycidylpyrrole-co-pyrrole), *Os* osphendione, *CHIT* chitosan, *ITO* indium tin-oxide, *SWCNT* single-walled carbon nanotubes.

**Table 2:** Recovery test in diluted human serum samples spiked with galactose.

Added (μM)	Found (μM)	Recovery (%)	RSD (%, *n* = 3)
10	8.6	86	5.2
100	90	90	7.9
1000	1010	101	4.5

## Supplementary Materials

The following are available online at https://www.mdpi.com/1424-8220/20/19/5632/s1, Figure S1: Representative linear sweep voltammograms of gold electrodes in 35 and 500 mM HCl solutions, Figure S2: SEM images of NPG-35 and NPG-500 in the 12 × 9.5 µm region, Figure S3: AFM images of an NPG electrode, Figure S4: Relationship between saccharide concentration and current density obtained from the voltammograms in Figure 2, Figure 3 and Figure 4, Figure S5: Cyclic voltammograms of NPG-500 in 0.1 M phosphate buffer solution of pH 6.0, 7.0 and 8.0 in the presence of 2 mM at galactose, Figure S6: Cyclic voltammograms of 10 mM galactose at NPG-35 in the presence of 0.1 M NaCl, Figure S7: Representative amperogram obtained at −0.1 V with successive additions of 0.2 mM AA, 2 mM mannose, 2 mM glucose, and 2 mM galactose, Figure S8: Cyclic voltammograms of NPG-500 in 0.5 M H_2_SO_4_ solution before and after seven amperometric measurements, and current densities of five different NPG-500 electrodes by amperometric measurements at –0.1 V.
