# Supplementary material for: Electrochemical Oxidation of Monosaccharides at Nanoporous Gold with Controlled Atomic Surface Orientation and Non-Enzymatic Galactose Sensing
Source: Sensors (Basel). 2020 Oct 1;20(19):5632. doi: 10.3390/s20195632 (PMC7582603; doi:10.3390/s20195632)
Supplement: Supplementary file 1 [file sensors-20-05632-s001.pdf]

—Supplementary Figures—

Electrochemical Oxidation of Monosaccharides at Nanoporous Gold with Controlled Atomic Surface Orientation and Non-enzymatic Galactose Sensing

Yasuhiro Mie *, Shizuka Katagai and Masiki Ikegami

Bioproduction Research Institute, National Institute of Advanced Industrial Science and Technology (AIST), Sapporo 062-8517, Japan; s.katagai@aist.go.jp (S.K.); m.ikegami@aist.go.jp (M.I.)

** Corresponding Author: Yasuhiro Mie*

Phone: +81-11-857-8913, Fax: +81-11-857-8915, Email: yasuhiro.mie@aist.go.jp

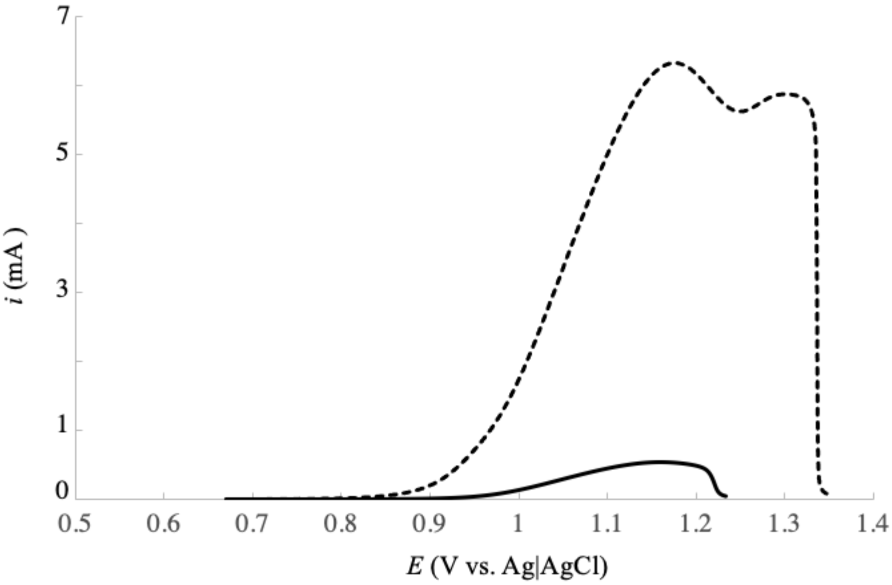

Figure S1. Representative linear sweep voltammograms of gold electrodes in 35 (solid line) and 500 mM (dashed line) HCl solutions at a scan rate of 50 mV s^{-1}. The passivation potentials were approximately 1.335 and 1.220 V in 500 and 35 mM HCl, respectively. Typical anodization was conducted at 1.310 and 1.185 V to prepare NPG-500 and NPG-35 for 1.5–3 min and 10–20 min, respectively.

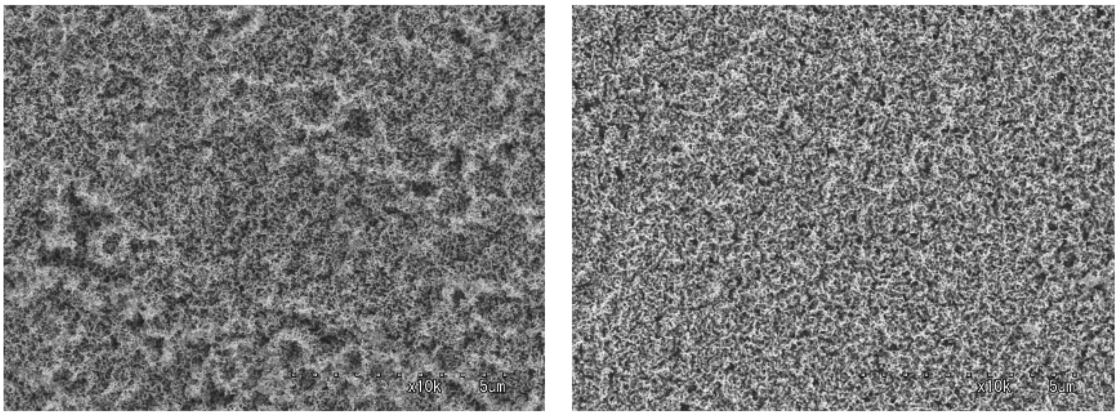

Figure S2. SEM images of NPG-35 (left) and NPG-500 (right) in the 12 × 9.5 μm region.

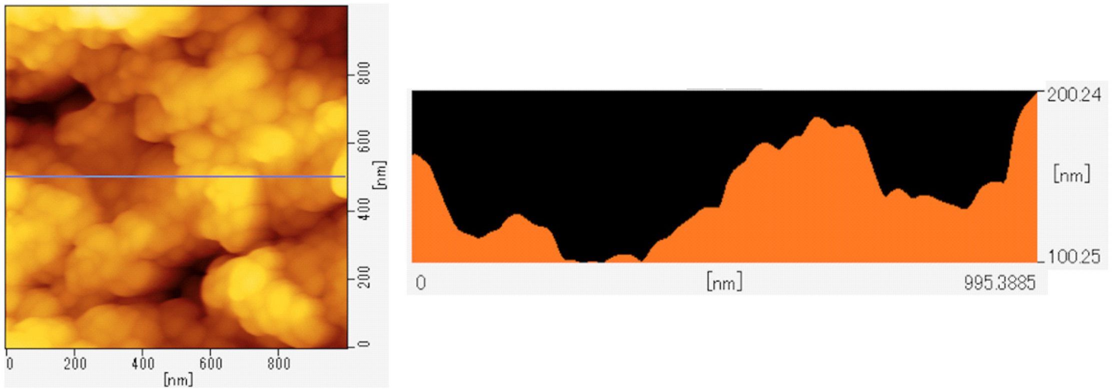

Figure S3. Atomic force microscopy (AFM) images of nanoporous gold (NPG) electrode in the x–y region of 1000 × 1000 nm square (left) and its depth profile (right) at the indicated line in the left image.

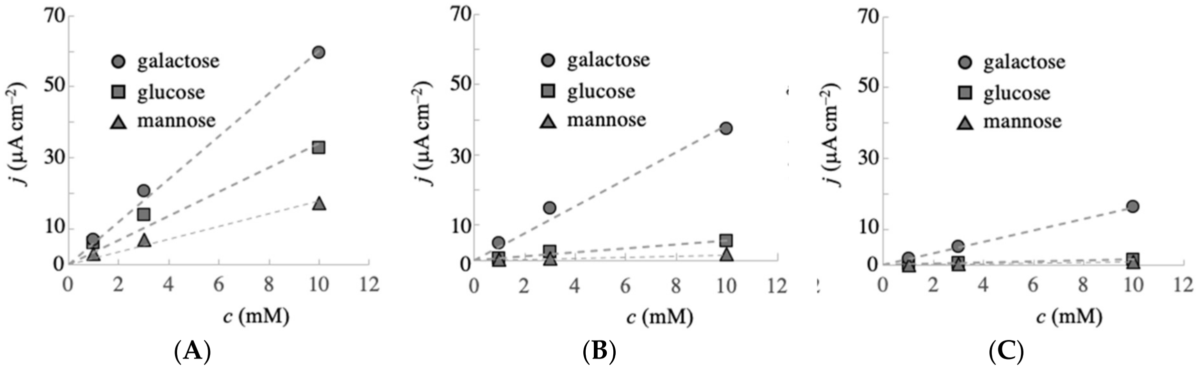

Figure S4. Relationship between saccharide concentration and current density obtained from the voltammograms in Figure 2A, Figure 3B, and Figure 4C, respectively, at –0.2 V. (**A**) and (**B**) were obtained in the absence of NaCl at NPG-500 and NPG-35, respectively, and (**C**) was in the presence of 0.1 M NaCl at NPG-500.

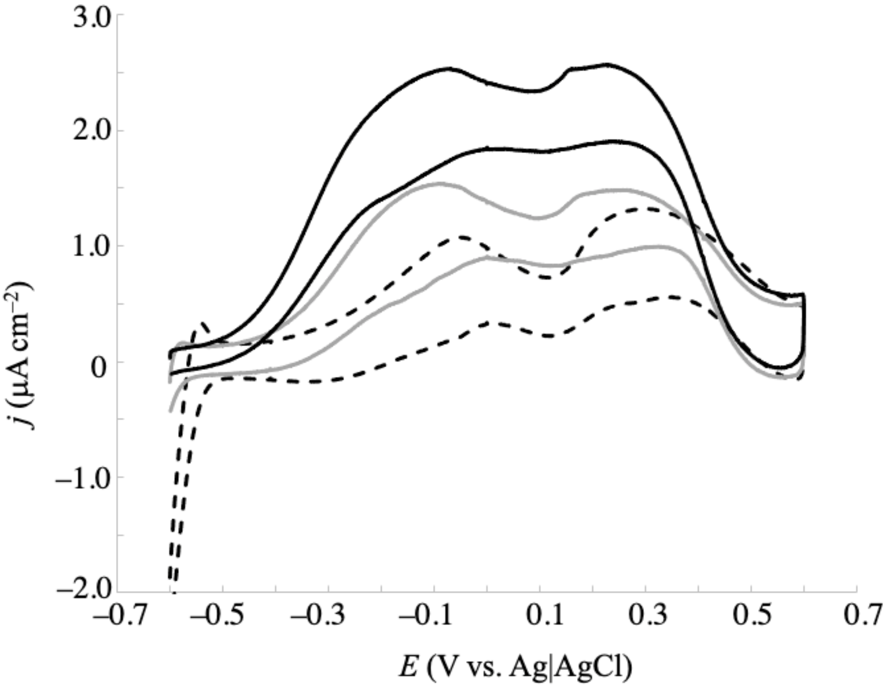

Figure S5. Cyclic voltammograms of NPG-500 in 0.1 M phosphate buffer solution of pH 6.0 (dashed black line), 7.0 (gray solid line), and 8.0 (black solid line) in the presence of 2 mM at galactose at a scan rate of 0.02 V s^{-1}.

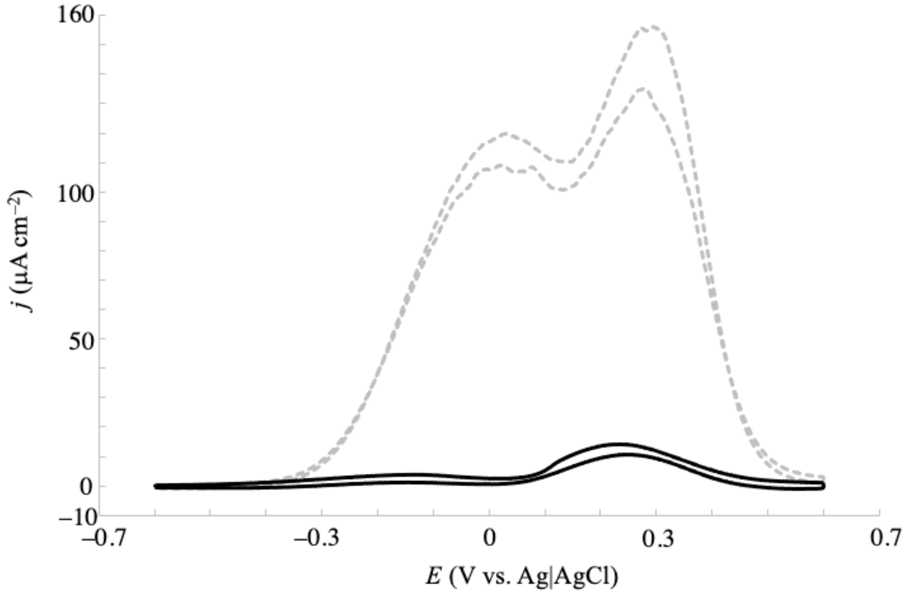

Figure S6. Cyclic voltammograms of 10 mM galactose at NPG-35 in the presence (black solid line) of 0.1 M NaCl in a 0.1 M phosphate buffer solution (pH 7.5) at a scan rate of 0.02 V s^{-1}, together with that in the absence of NaCl (gray dotted line).

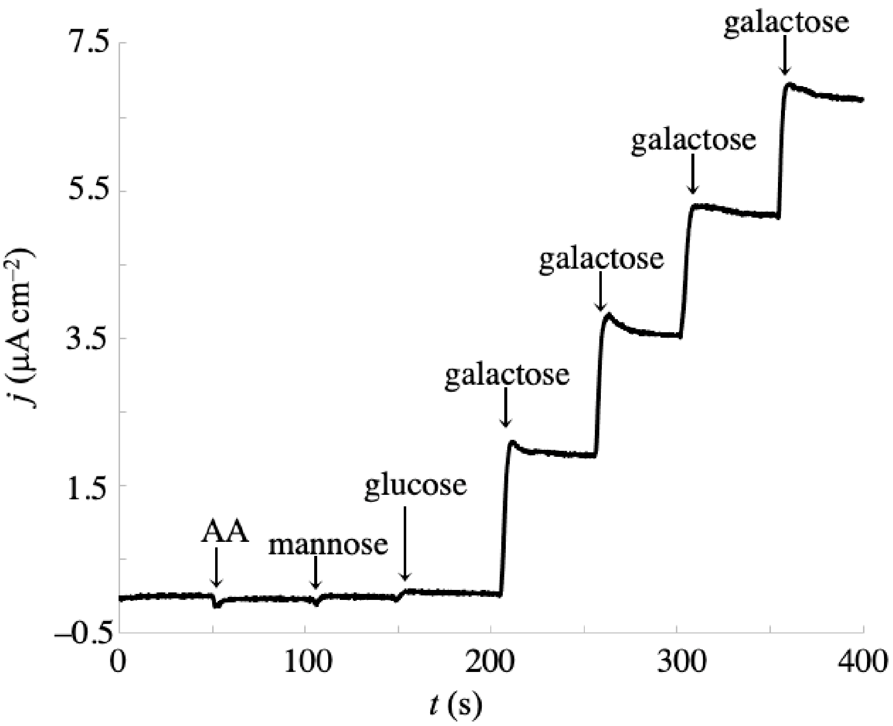

Figure S7. Representative amperogram obtained at −0.1 V in a phosphate buffer (pH 7.5) containing 0.1 M NaCl, with successive additions of 0.2 mM ascorbic acid (AA), 2 mM mannose, 2 mM glucose, and 2 mM galactose at 100 s intervals.

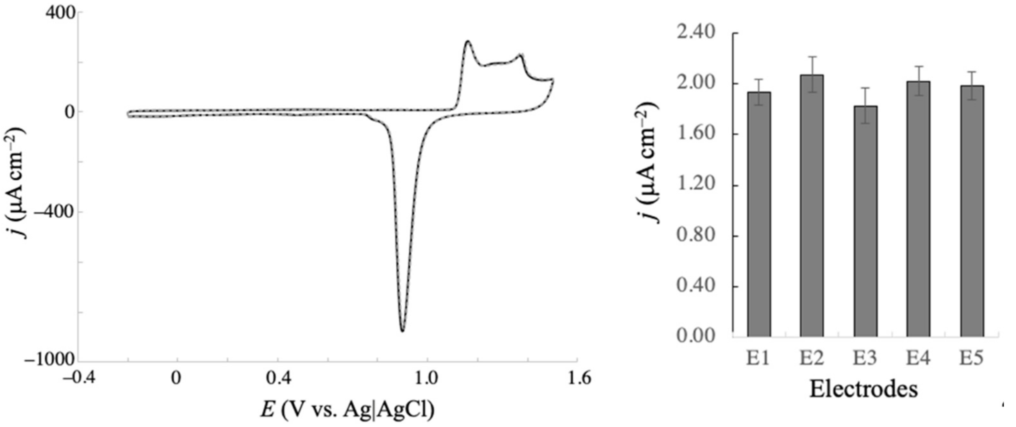

(**A**) (**B**)

Figure S8. (**A**) Cyclic voltammograms of NPG-500 in 0.5 M H_2SO_4 solution before (solid black line) and after (dashed gray line) seven amperometric measurements for galactose at a scan rate of 0.1 V s^{-1}. (**B**) Current densities of five different NPG-500 electrodes by amperometric measurements at –0.1 V in 0.1 M phosphate buffer (pH 7.5) containing 2 mM galactose and 0.1 M NaCl. Each electrode was used for triplicate determinations, and an error bar indicates the standard deviations.
